# Multiprotein collagen/keratin hydrogel promoted myogenesis and angiogenesis of injured skeletal muscles in a mouse model

**DOI:** 10.1186/s12896-024-00847-4

**Published:** 2024-04-26

**Authors:** Atieh Rezaei Namjoo, Ayla Hassani, Hassan Amini, Fateme Nazaryabrbekoh, Sepideh Saghati, Mohammad Ali Ebrahimi Saadatlou, Ali Baradar Khoshfetrat, Nafiseh Didar Khosrowshahi, Reza Rahbarghazi

**Affiliations:** 1 Stem Cell Research Center, Tabriz University of Medical Sciences, Imam Reza St, Golgasht St, Tabriz, Iran; 2 Chemical Engineering Faculty, Sahand University of Technology, Tabriz, 51335-1996 Iran; 3 Student Research Committee, Tabriz University of Medical Sciences, Tabriz, Iran; 4 Department of General and Vascular Surgery, Faculty of Medicine, Tabriz University of Medical Sciences, Tabriz, Iran; 5 Department of Tissue Engineering, Faculty of Advanced Medical Sciences, Tabriz University of Medical Sciences, Tabriz, Iran; 6 Department of Basic Sciences, College of Veterinary Medicine, Tabriz Branch, Islamic Azad University, Tabriz, Iran; 7 Department of Applied Cell Sciences, Faculty of Advanced Medical Sciences, Tabriz University of Medical Sciences, Tabriz, Iran

**Keywords:** Collagen/keratin hydrogel, Myogenic properties, Volumetric loss, Mouse, Tissue engineering

## Abstract

Volumetric loss is one of the challenging issues in muscle tissue structure that causes *functio laesa*. Tissue engineering of muscle tissue using suitable hydrogels is an alternative to restoring the physiological properties of the injured area. Here, myogenic properties of type I collagen (0.5%) and keratin (0.5%) were investigated in a mouse model of biceps femoris injury. Using FTIR, gelation time, and rheological analysis, the physicochemical properties of the collagen (Col)/Keratin scaffold were analyzed. Mouse C2C12 myoblast-laden Col/Keratin hydrogels were injected into the injury site and histological examination plus western blotting were performed to measure myogenic potential after 15 days. FTIR indicated an appropriate interaction between keratin and collagen. The blend of Col/Keratin delayed gelation time when compared to the collagen alone group. Rheological analysis revealed decreased stiffening in blended Col/Keratin hydrogel which is favorable for the extrudability of the hydrogel. Transplantation of C2C12 myoblast-laden Col/Keratin hydrogel to injured muscle tissues led to the formation of newly generated myofibers compared to cell-free hydrogel and collagen groups (*p* < 0.05). In the C2C12 myoblast-laden Col/Keratin group, a low number of CD31^+^ cells with minimum inflammatory cells was evident. Western blotting indicated the promotion of MyoD in mice that received cell-laden Col/Keratin hydrogel compared to the other groups (*p* < 0.05). Despite the increase of the myosin cell-laden Col/Keratin hydrogel group, no significant differences were obtained related to other groups (*p* > 0.05). The blend of Col/Keratin loaded with myoblasts provides a suitable myogenic platform for the alleviation of injured muscle tissue.

**Supplementary Information:**

The online version contains supplementary material available at 10.1186/s12896-024-00847-4.

## Introduction

The muscular tissue is composed of numerous multi-nucleated aligned myocytes supported by the vascular bed and neural networks [1]. The existence of the ternary connective tissue layer, epimysium, perimysium, and endomysium, provides a specific niche for the preservation of muscle cells [1, 2]. Besides, these layers act as supporting basal lamina for the maintenance of muscle progenitor cells, namely satellite cells [3]. It is suggested that satellite cells are involved in muscle regeneration in response to several insulting conditions, leading these cells to exit from quiescent status, enter proliferation, and subsequently differentiate into mature myocytes [4]. Despite the regenerative potential of satellite cells, extensive and bulk injuries to the muscle mass lead to abnormal extracellular matrix (ECM) production, and fibrotic changes [5]. At present, the replacement of fibrotic tissues with highly vascularized and innervated grafts from autologous or allogenic sources is touted as an available therapeutic modality [6–8]. However, the possibility of immune reaction, infection transmission, volunteers, and post-surgical complications are the main issues associated with the allogenic transplantation of muscle grafts [9, 10]. In this regard, recent years have witnessed advancements in the development of tissue engineering approaches to circumvent these limitations [11, 12]. Tissue engineering is a multidisciplinary scientific branch to creating optimal therapeutic modalities using certain biomaterials, specific cell types, and diverse growth factors [13]. In contrast to whole-cell therapy, tissue engineering tries to increase the regenerative potential of transplant cells by providing an ECM-like platform, known also as a scaffold, to regulate and orient cell bioactivity toward specific lineages [13, 14]. To this end, many researchers have examined the regenerative potential of scaffolds composed of natural, synthetic, and semi-synthetic substrates [15–17]. As their names suggest, natural scaffolds are commonly fabricated from organic substrates with high-rate biocompatibility and degradation rates [18]. Despite these advantages, inappropriate physicochemical properties and sensitivity to enzymatic activity limit their application [19]. Therefore, attempts should be directed toward the development of composites with distinct formulations that can circumvent the above-mentioned limitations [20, 21]. Among different scaffold types, injectable hydrogels are at the center of attention because of the introduction to the injured site with minimally invasive manipulations [22, 23]. Of note, weak mechanical properties, fragile structure, and rapid degradation are the main problems associated with the application of hydrogel in solid tissues such as muscular tissue [24]. The simultaneous application of substrates with similar properties is touted as an efficient way to achieve better regenerative outcomes [25, 26]. Keratin is a cysteine-rich protein with significant biocompatibility and prolonged durability and has been selected as a suitable supporting substrate for the fabrication of hybrid hydrogels [27]. It was suggested that keratin is an eligible substrate to provide several motifs and a favorable 3D microenvironment for cell attachment and expansion [27, 28]. Keratin-based hydrogels can benefit from proper mechanical stability keratin-based hydrogels have been increased in the tissue engineering of solid tissue such as muscles [29–31]. Herein, we tried to assess the myogenic properties of hydrogel composed of type I Col and keratin in a mouse model of biceps femoris injury.

## Materials and methods

### Keratin extraction

To this end, human head hair samples were collected from local barbershops and keratin content extracted based on the previously published protocols [30, 32]. In short, 200 g human head hair samples were washed using phosphate-buffered saline (PBS) to exclude debris and contamination followed by putting them in a 37˚C oven to dry. To remove lipid content, samples were incubated in chloroform (Cat no: 1.02445.2500; Merck)/methanol (Cat no: 1.6009.2500; Merck) solution (2:1 v/v) for 24 h. After that, samples were immersed in 20% v/v peracetic acid (PAA; Cat no: Cat No: 1,072,221,000; Merck) solution and kept at 40˚C for 24 h and then washed with PBS for complete acid solution removal. The procedure was continued with the incubation of hair mass inside the 100 mM Tris-based (Cat No: 1,083,820,100; Merck) solution for 2 h. The resulting protein solution was then separated using 2 mm-pore-sized stainless steel meshes. Protein samples were centrifuged at 10,000 rpm, dialyzed using 12 kD cut off the membrane (Cat no: D0530; Sigma-Aldrich), neutralized, lyophilized, and stored at -80˚C until use.

### Hydrogel preparation

About 0.125 g of type I Col powder (Molecular weight: Mw116 kDa; SBPE Company; Iran) was dissolved in 25 ml acetic acid (0.1 M) to obtain a 0.5% Col solution. After neutralization with sodium hydroxide solution (Cat no: 109141.1000; Merck), 0.06 g keratin powder was added to the 6 ml Col solution (0.5%) to prepare hybrid Col (0.5%)/Keratin (0.5%) hydrogel. The mixture of Col/Keratin was sterilized using a chloroform solution. Gelation phenomenon occurs through thermal crosslinking process.

### Physicochemical analyses

#### Fourier transform infrared (FT-IR)

FTIR analysis was performed using Bruker Tensor 27 Germany. Col, Keratin, and a combination of Col/Keratin were mixed with potassium bromide (KBr) powder to provide a KBr pellet. Ultimately, a spectrum of each sample was acquired with a range of wavenumber between 250 and 4500 cm^− 1^.

#### Gelation time

Here, we measured the gelation time of Col and Col/Keratin hydrogels using the inverted test tube technique. For this purpose, 1 ml solution was poured into vials and immersed in PBS followed by incubation at 37 °C. The gelation time was recorded as the solution inside the vial never flowed by tilting the vial, while the vial has been tilted every 5 min. Every sample was examined in duplicate.

#### Rheological properties

To explore the viscosity rate, the rheological properties of fabricated hydrogels were examined via Anton Paar 301 rheometer. All samples were fixed between two plates of the rheometer (interval of 0.8 mm) at an ambient temperature of 37 °C. First, the strain sweep was examined in the range between 0.1 and 100% at a constant frequency of 1 Hz to detect the linear viscoelastic range. Then, the frequency sweep of the samples was determined between ranges of 0.1–100 1/s at a constant strain of 1% (which was obtained through a strain sweep test as a critical strain point). Finally, the time sweep was detected at a frequency of 1 Hz and strain of 1% for the 2500s. Moreover, the shear viscosity of samples separately was obtained through a shear rate range of 0.1 to 100 s^− 1^ to investigate the shear thinning property of fabricated hydrogels.

### Culture of mouse myoblast cell line C2C12

C2C12 cells were purchased from National Iranian Cell Bank (Tehran, Iran). Cells were expanded in a high-glucose content DMEM culture medium (Gibco) supplemented with 10% fetal bovine serum (Cat no: F7524; Sigma-Aldrich) and 1% Penicillin-Streptomycin (Cat no: BI1203; BIO-IDEA). Cells were cultured under standard conditions with 5% CO_2_ and 95% humidity at 37˚C. Cells at 70–80% confluence were detached using a 0.25% Trypsin-EDTA solution (Cat no: BI-1602; BIO-IDEA). In this study, we used C2C12 cells at passages between 3 and 6.

### Induction of mouse model of muscle injury

**Fig. 1:**
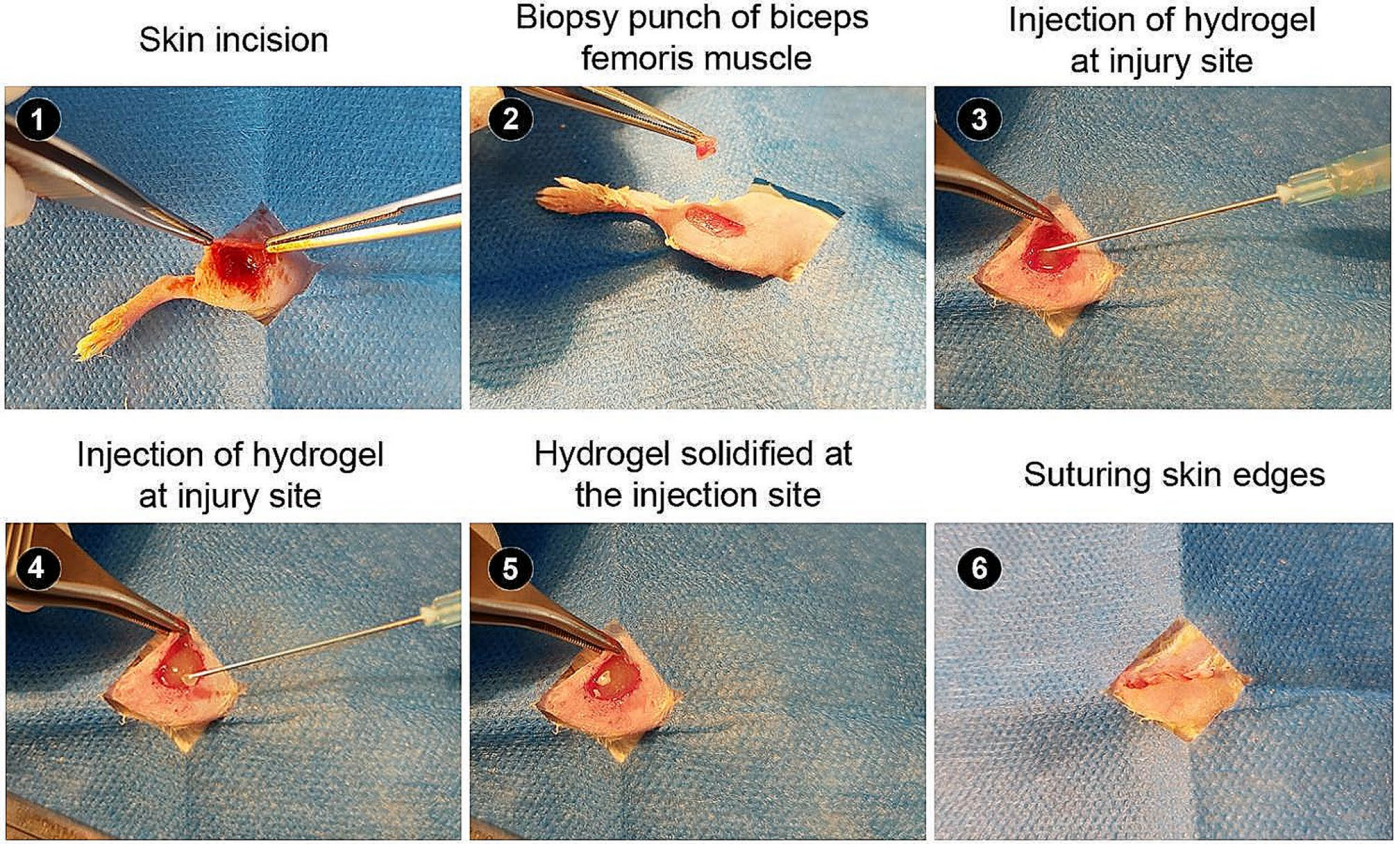
Surgical procedure for the induction of volumetric muscle loss in a mouse model after biceps femoris muscle injury and transplantation of myoblast-laden Col/Keratin hydrogel. After shaving and disinfection, a surgical incision was made to get access to the biceps femoris muscle mass. For volumetric muscle loss, segments of skeletal muscle surgical were removed surgically. The process followed by the injection of Col, and Col/Keratin hydrogels with or without the myoblasts. After being solidified, the incision line was carefully sutured and the animals were kept for 15 days

All phases of this study were approved by the Local Ethics Committee of Tabriz University of Medical Sciences. The mice were obtained from the animal house of Tabriz University of Medical Sciences. Twenty-24 male BALB/c mice (5-week-old; 20–25 g) were kept at standard conditions with free access to water and chewing food. To induce muscle injury, animals were deeply anesthetized using a combination of Ketamine (90 mg/kbw)-Xylazine (10 mg/kbw). After that, the cutaneous tissue of the thigh area was shaved and sterilized. Then, an incision was made on the posterior of the thigh area skin to expose the muscular layer (Fig. 1). The procedure was continued by the separation of superficial fascia from muscle mass and injury was created by resecting a segment (about 4–5 mm) of the biceps femoris posterior muscle. In treatment groups, 0.5 ml hydrogel was injected into the defect sites. After the completion of the injection, the facial layer and skin were sutured using 6 − 0 Vicryl and 5 − 0 prolene strings. Mice received analgesic treatment and antibiotics. In this study, animals were randomly allocated into four groups (each in 6) as follows; Col, Col + Cell, Col/Keratin, and Col/Keratin + Cells. In groups that received cells, an approximate number of 1 × 10^6^ C2C12 cells per 0.5 ml hydrogel was injected into the target site. In cell-free groups, normal saline was used as a vehicle.

### Histological examination

Fifteen days post-transplantation, mice were euthanized using an overdose of Ketamine and xylazine. Samples were carefully taken and divided into two parts for proteomic analysis and histological examination. To study the general feature of injured muscles after injection of hydrogels, samples were fixed in a 10% neutral buffered formalin solution. After PBS washes, samples were dehydrated and paraffin-embedded. In the next steps, 5-µm thick slides were prepared and stained using a Hematoxylin-Eosin solution. For monitoring the angiogenesis procedure within the transplanted hydrogels, immunohistochemistry (IHC) staining was performed. Prepared slides were incubated in sodium citrate (pH 6.5) for 20 min for antigen retrieval. To inhibit endogenous peroxidase activity, samples were exposed to 3% H_2_O_2_ for 10–15 min followed by blocking in 3% goat serum solution. After that, slides were treated with mouse anti-human CD31 (Dako; dilution 1:100) for 24 h at 4˚C. Following three-time PBS washes, HRP conjugated secondary antibody was used. DAB was used as a chromogenic agent.

### Western blotting

Tissue samples were collected from the biceps femoris posterior muscles. Total protein content was extracted using RIPA protein lysis buffer containing an anti-protease cocktail followed by centrifugation at 12,000 rpm at 4 °C for 20 min. Samples were electrophoresed in 10% SDS-PAGE and transferred to the PVDF membrane. For blocking PVDF membranes were maintained in the presence of 1% bovine serum albumin for 1 h at RT. For monitoring myogenic properties, protein levels of MyoD1 and anti-heavy chain myosin were measured by the incubation of membranes in a solution containing anti-MyoD1 (Cat no: ab64159; Abcam), and anti-heavy chain myosin (Cat no: ab50967; Abcam) antibodies at 4˚C overnight. To reduce background, membranes were washed three times in PBST solution each in 10 min. Immunoreactive bands were detected using an ECL solution and the density rate was calculated using ImageJ software (NIH).

### Statistical analysis

Data are represented as mean ± SD and analyzed using One-way ANOVA with Tukey post hoc (GraphPad Prism, version 8). All experiments were done in triplicate otherwise mentioned. P values below 0.05 were considered statistically significant.

## Results

### FT-IR analysis

**Fig. 2:**
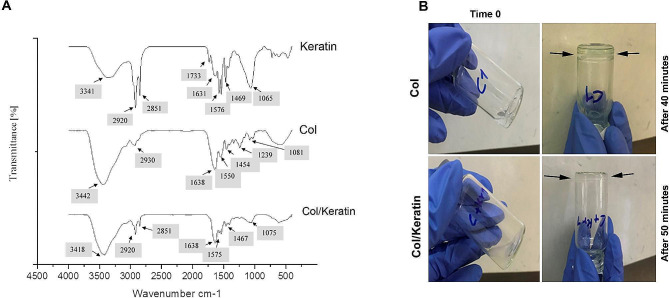
FTIR analysis of Col, Keratin, and Col/Keratin hydrogels (**A**). Data confirmed that the mixture of Col and Keratin yielded prominent peak shifting in the amide I area from 3442 cm^− 1^ to 3418 cm^− 1^, amide II area from 1550 cm^− 1^ to 1575 cm^− 1^, C-H bending vibration area from 1454 cm^− 1^ to 1467 cm^− 1^, and the emergence of C-H symmetric stretching vibration at 2851 cm^− 1^. Monitoring gelation time in Col (0.5% w/v)/(0.5% w/v)Keratin hydrogel compared to the (0.5% w/v) Col hydrogel alone (**B**). The addition of Keratin to Col solution prologned the gelation time from 40 to 50 min

The FT-IR spectrum of the Col, keratin, and Col/Keratin hydrogel is illustrated in Fig. 2A. In Col, N-H stretching band as the amide I group appeared at 3442 cm^− 1^. The band at 2930 cm^− 1^ correlates with C-H asymmetrical stretching vibrations of the methyl group. Besides, C-H bending vibration was also observed at 1450 cm^− 1^ [33]. IR bands at 1638, 1550, and 1239 cm^− 1^ are associated with C = O stretching vibrations for amide I, N-H bending to C-N stretching vibrations for amide II, and C-N stretching vibrations for amide III [34]. C-O and C-O-C stretching vibration of carbohydrate subunits appeared at 1081 cm^− 1^ [35]. In the keratin group, the broad peak at 3341 cm^− 1^ is associated with N-H stretching vibration; resulting in the formation of the amide I band (Fig. 2A). The peaks at 2920 cm^− 1^ and 2851 cm^− 1^ correspond to C-H asymmetrical and symmetrical stretching of methyl and methylene groups. Additionally, C = O stretching vibration at 1631 cm^− 1^ is related to β-sheets of polypeptide structure. Furthermore, the peak at 1576 cm^− 1^ is associated with N-H bending vibration and C-N stretching vibration for the Amide II group. Typically, the signals of polypeptide structure in the range of amide III appear weaker than in the range of the amide I band [36]. A broad sharp peak is also apparent at 1080 cm^− 1^ associated with the S–O stretching vibration of cysteine-S-sulfonate residues [37, 38] The emergence of the carbonyl group (C = O) band at 1733 cm^− 1^ is possibly due to the partial residue oxidation of keratin in the presence of peracetic acid during the extraction steps [39]. The combination of Col with Keratin leads to prominent peak shifting in the amide I area from 3442 cm^− 1^ to 3418 cm^− 1^, amide II area from 1550 cm^− 1^ to 1575 cm^− 1^, C-H bending vibration area from 1454 cm^− 1^ to 1467 cm^− 1^, and the emergence of C-H symmetric stretching vibration at 2851 cm^− 1^ (Fig. 2A). The addition of keratin to Col solution caused peak shifting in keratin structure within the amide I area from 1631 cm^− 1^ to 1638 cm^− 1^ and reduced the peak absorption and peak shifting at 1065 cm^− 1^, resulting in enhanced peak absorption and peak shifting at the amide I area. Taken together, the incorporation of keratin into Col has an impact on the emerged IR bonds of structural amide groups, which are integrated into hydrogen bond formation [34]. These features show an appropriate interaction between keratin and Col.

### Gelation time

The gelation of Col/Keratin hydrogel was monitored at 37˚C and a pH value of 7.8 using a tilting tube method every 5 min (Fig. 2B). Based on our data, Col hydrogel gelled after 40 min without any flow through the tilting tube. Data indicated that the gelation of Col/Keratin hydrogel occurs after 50 min. The mixture of Col/Keratin exhibited an opaque appearance with a softer structure compared to the Col group. It seems that the addition of keratin to Col solution can affect hydrogel stiffness possibly via the reduction of hydrogen bonds.

### Rheological behavior

**Fig. 3:**
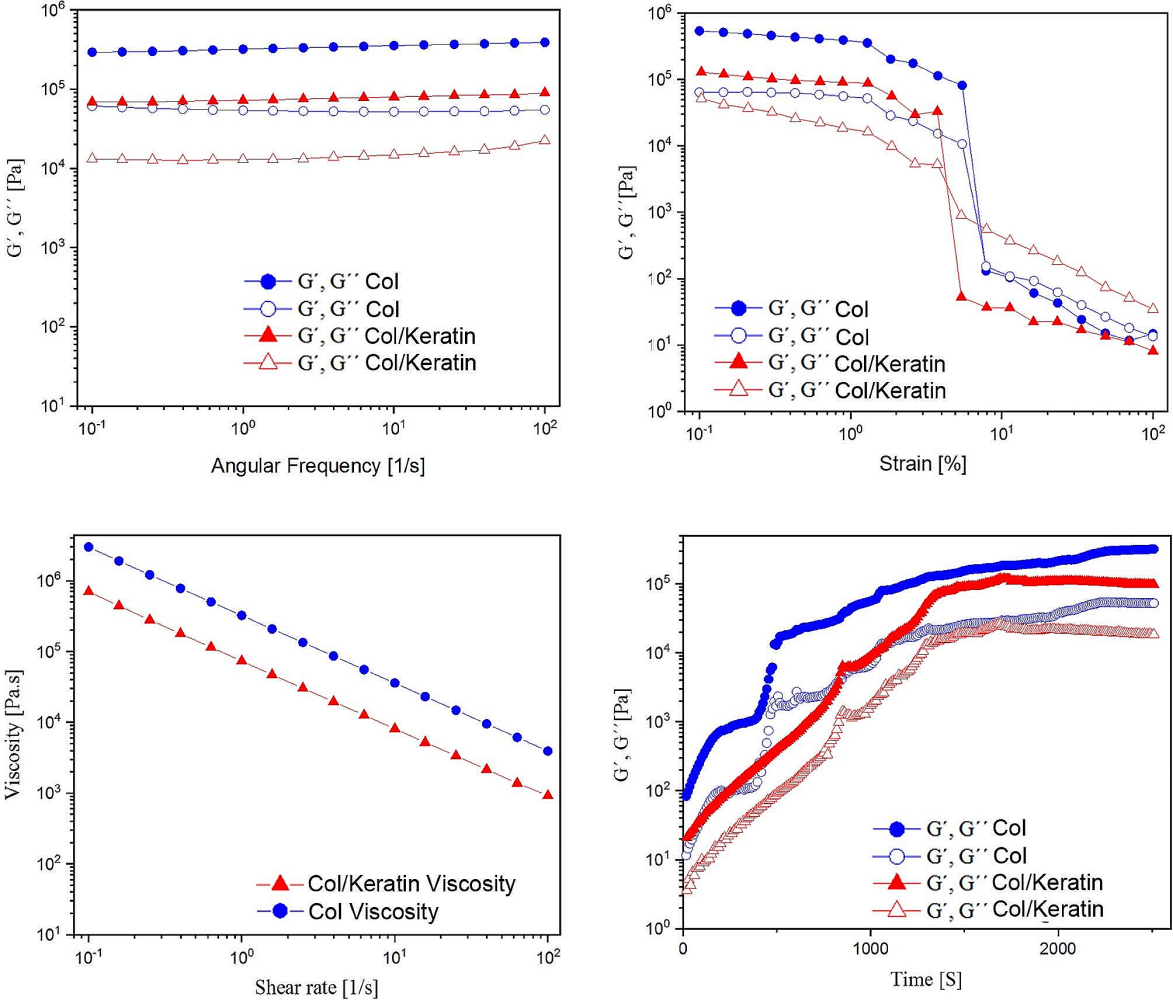
Rheological analysis of Col and Col/Keratin hydrogels. Data indicated Col hydrogel alone exhibited a higher viscosity and stiffness rate compared to the Col/Keratin group

Rheological characterization was performed to discover the injectability, stability, and blending duration (Fig. 3) [40]. The time sweep measurement exhibited consistent and mild crosslinking of both hydrogels within 40 min considering the surpassing of G´ from G´´ at the beginning of a blending process, relatively similar to tube inversion results. Additionally, a shear-thinning property, an essential feature that indicates the hydrogel injectability, was observed. We found viscosity reduction by increasing the shear rate within 0.1–100 1/s. However, Col hydrogel alone exhibited a higher viscosity and stiffness rate compared to Col/Keratin group. Regarding strain sweep, G´, which was higher than G´´ in the linear viscoelastic area, indicates gel-like behavior and a stable network of hydrogels. The bigger G´ of Col hydrogel was obtained, indicating higher blending density (Fig. 3). Due to the frequency sweep in a linear viscoelastic area within 0.1–100 1/s, both hydrogels appeared frequency-independent behavior associated with excellent blending density and stable network formation. These values were high in the Col hydrogel compared to Col/Keratin group.

### Combination of keratin and Col promoted myogenesis

**Fig. 4:**
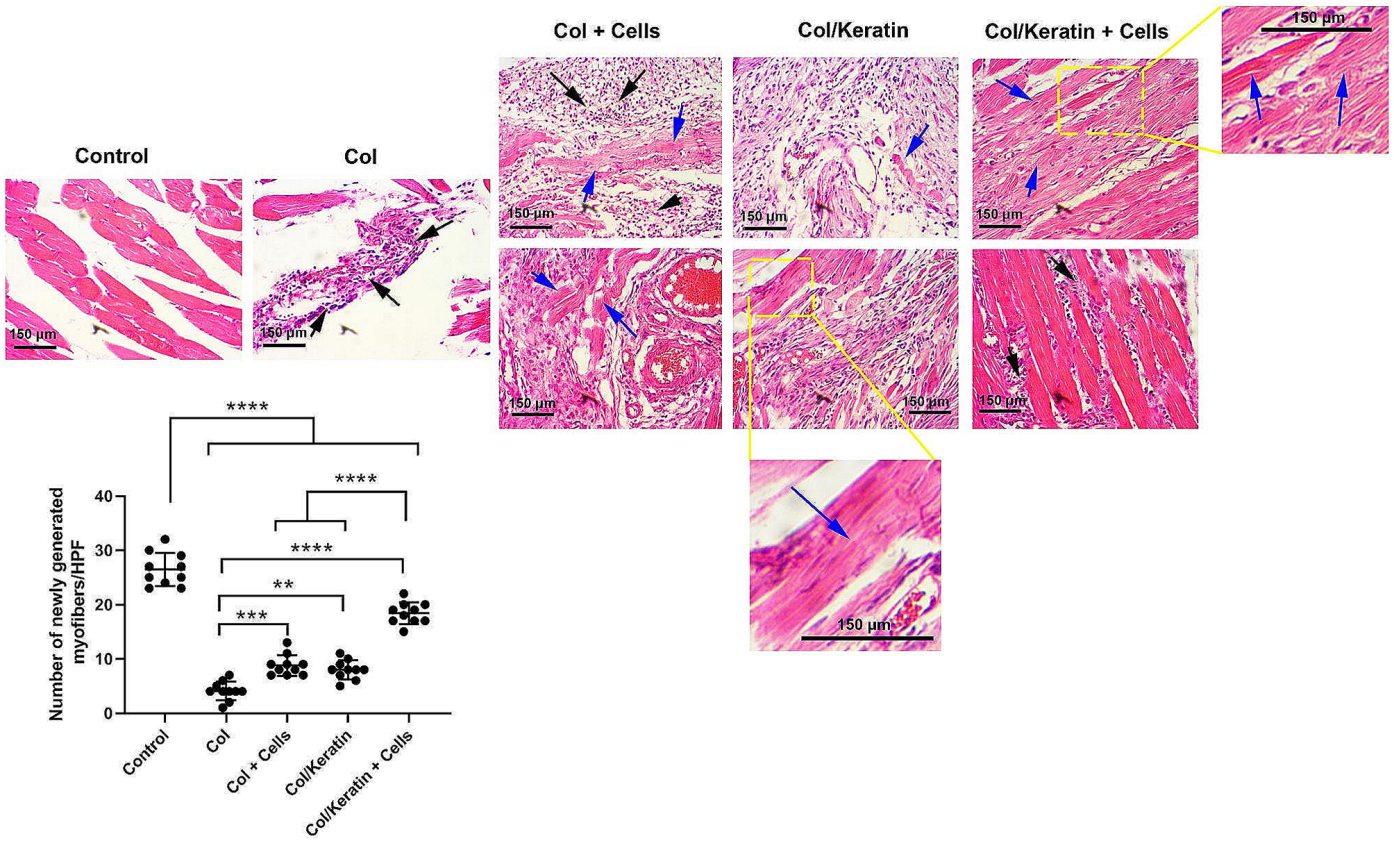
H & E staining of injured muscle tissue after transplantation of hydrogel. Mice were allocated into Col, Col + Cells, Col/Keratin, and Col/Keratin + Cells groups. Bright-field imaging indicated that in the group that received Col hydrogel alone, numerous inflammatory cells were recruited to the injury site (black arrows), leading to the digestion and scavenging of hydrogel remnants. These features were less in other groups that received the combination of cells with hydrogel. In Col + Cells, inflammatory cells (black arrows) are at the periphery of newly generated myofibers (blue arrows). The myofibers are not aligned in a regular pattern. In the Col + keratin group, the intensity of immune cells was reduced and newly generated myofibers exhibited a more aligned pattern (blue arrows). Maximum myofiber density was achieved in the group with the transplantation of Col/Keratin and Cells. The area was filled with aligned newly generated myofibers (blue arrows) and few immune cells can be evident in intermyocyte space (black arrows). Data analysis revealed the maximum myofiber formation in the Col/Keratin + Cells group compared to the other groups (10 high-power fields). One-way ANOVA analysis with Tukey test. **p < 0.01; ***p < 0.001; and ****p < 0.0001

H&E staining of the biceps femoris indicated the generation of *de novo* muscle fibers in mice that received the combination of cells and hydrogels after 15 days (Fig. 4). In the group that received Col hydrogel alone, numerous immune cells were recruited into the injured sites (black arrows), leading to the degradation of transplanted hydrogel. The presence of immune cells in the defect area without newly generated myofibers indicated the failure in the activity of the endogenous reparative system to restore muscle function. In the Col + Cell group, few newly generated myofibers (blue arrows) can be detected at the injection sites which are surrounded by numerous immune cells (black arrows) (Fig. 4). Along with these changes, hyperemia is also evident. Similarly, a few newly generated myofibers were obtained in injured mice that received the combination of Col/Keratin. Finally, it was notified that the injection of encapsulated cells with Col/Keratin hydrogel caused clearly to the generation of myofibers. Bright-field imaging revealed the existence of immune cells (black arrows) at the periphery of myofibers which is possibly due to the scavenging of the remnants of injected hydrogel or local tissue debris. Notably, transplantation of myoblast-loaded Col/Keratin hydrogel resulted in a significant number of myofibers compared to the other groups (*p* < 0.05). Based on the data, cell-free Col hydrogel yielded the least number of newly generated myofibers compared to the other groups. Interestingly, non-significant differences were obtained in terms of myofibers between Col + Cells and Col/Keratin groups (*p* > 0.05). These data indicated that the co-transplantation of C2C12 cells with the combination of Col and keratin can promote myogenesis in muscle injury sites.

### Monitoring angiogenesis capacity

**Fig. 5:**
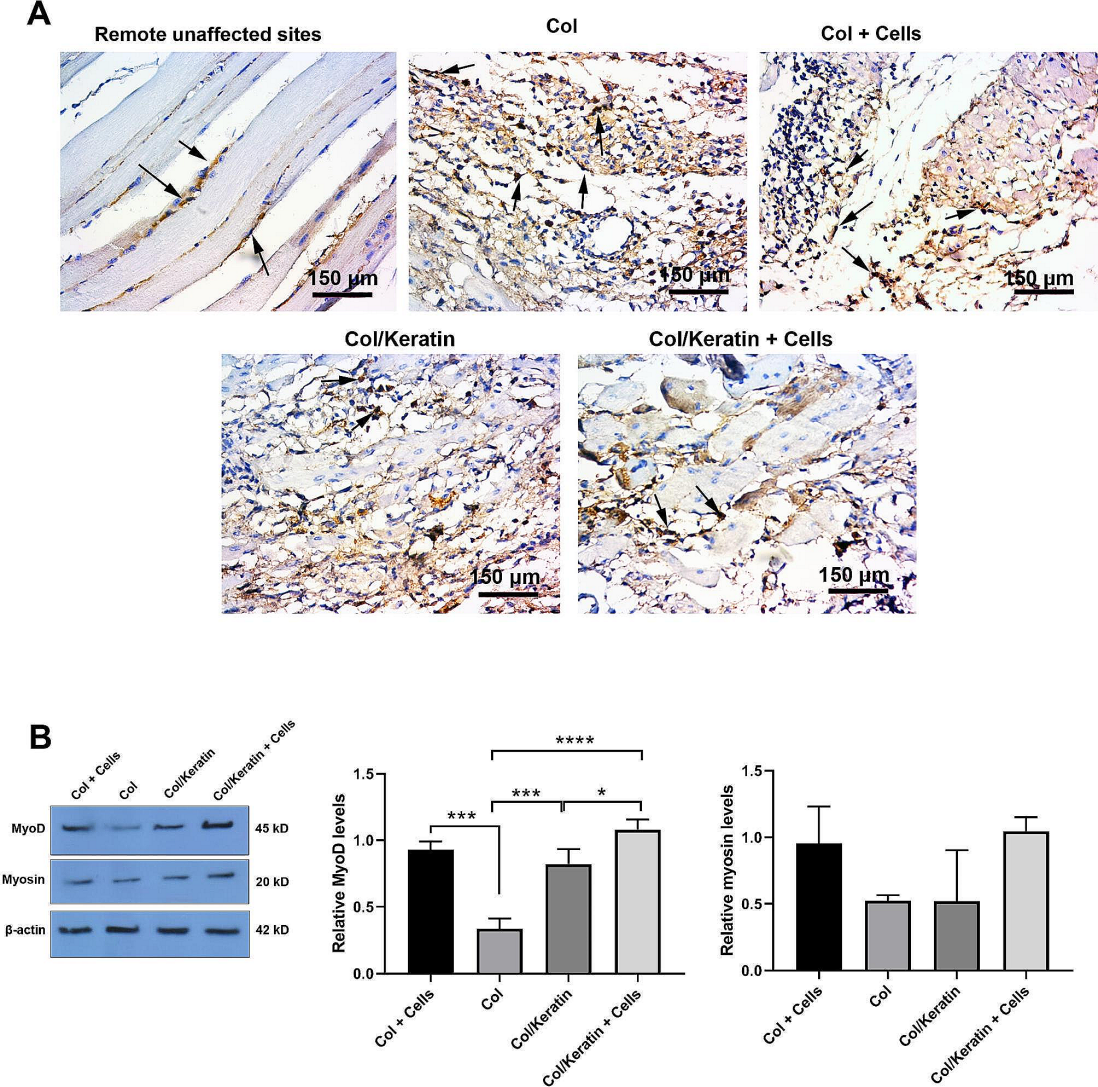
IHC staining of CD31 positive cells in different groups after transplantation of hydrogel (**A**). CD31^+^ cells are indicated using black arrows. In the control group, CD31^+^ cells with flattened morphology are juxtaposed with normal myocytes in the muscular tissue. In the Col group, numerous CD31^+^ cells can be detected within the injection site without a regular pattern, indicating the promotion of angiogenesis due to immunological response. Data indicated more aligned CD31^+^ cells in the Col + Cells group. The intensity and number of CD31^+^ cells were reduced in Col/Keratin and Col/Keratin + Cells groups. CD31^+^ cells can be detected at the periphery of newly generated myofiber in the Col/Keratin + Cells group. Westernblotting analysis of MyoD and myosin (**B**; *n* = 3). Data revealed the induction of MyoD in Col + Cells and Col/kertain + Cells groups, indicating the maturation of muscle progenitor cells. Despite the increase of myosin in the Col/Kertain + Cells group, the differences were statistically non-significant. One-way ANOVA analysis with Tukey test. *p < 0.05; ***p < 0.001; and ****p < 0.0001

IHC staining of CD31^+^ cells was done to monitor angiogenesis potential within the hydrogels after 15 days after transplantation at the site of muscle tissue injury (Fig. 5A). Based on the data, numerous recruited immune cells plus CD31^+^ cells are evident within the Col hydrogel matrix (black arrows). The increase of CD31^+^ cells would be possibly related to an active inflammatory response to degrading the injected Col mass. The number of CD31^+^ cells was reduced in injured mice that received the combination of Col plus C2C12 cells. ECs cells can be detected at the periphery of newly generated myofibers (black arrows) (Fig. 5A). In the Col/Keratin group, the number of generated myofibers was increased along with the reduction of CD31^+^ cells and pro-inflammatory response compared to the Col and Col + Cell groups. We found the minimum CD31^+^ cells in the injury sites after injection of Col/Keratin + Cells, indicating accelerate regeneration rate and restoration of injured sites with the cells that are morphologically similar to the natural myocytes (black arrows).

### Col/Keratin hydrogel plus C2C12 myoblasts increased myocyte differentiation

To assess whether the injection of Col/Keratin hydrogel plus C2C12 myoblasts can affect C2C12 differentiation and functional capacity, protein levels of MyoD and myosin were measured using western blotting (Fig. 5B). Data revealed the minimum levels of MyoD protein in mice that received Col hydrogel alone compared to the other groups (*p* < 0.05). Based on the data the combination of Col with keratin increased significantly local MyoD levels related to the Col hydrogel group (*p* < 0.001; Fig. 5B). Although co-transplantation of C2C12 cells with Col hydrogel increased MyoD levels these changes did not reach statistically significant levels. Notably, co-administration of C2C12 cells along with Col/Keratin yielded maximum levels of MyoD in comparison with Col/Keratin and Col groups. It seems that simultaneous supplementation of the Col/Keratin hydrogel with myoblasts can increase is an effective way to increase local levels of MyoD factors at the site of injection. Regarding protein levels of myosin, statistically non-significant differences were obtained. Despite the increase of myosin levels in Col + Cells and Col/Keratin + Cells groups, these changes did not yield significant differences (*p* > 0.05) (Fig. 5B). These data demonstrated that the combination of Col and keratin is an appropriate substrate to support the myogenic potential of myoblasts after injection into the injury sites within the muscular tissue.

## Discussion

Muscle injuries and pathologies are challenging issues in the clinical setting. Muscle tissue engineering aims to foster the regeneration of injured sites by combining biology, chemistry, and engineering tools [41]. Hydrogels, composed of synthetic and natural substrates, have been commonly used as 3D cell culture platforms to relatively mimic the native muscular tissue microenvironment and healing process [42]. Along with various 3D in vitro models, the application of several animal models, especially rodents, can provide invaluable in vivo platforms to elucidate the underlying mechanisms involved in the regeneration of muscular tissue after transplantation of engineered grafts into the site of injury [43].

**Fig. 6:**
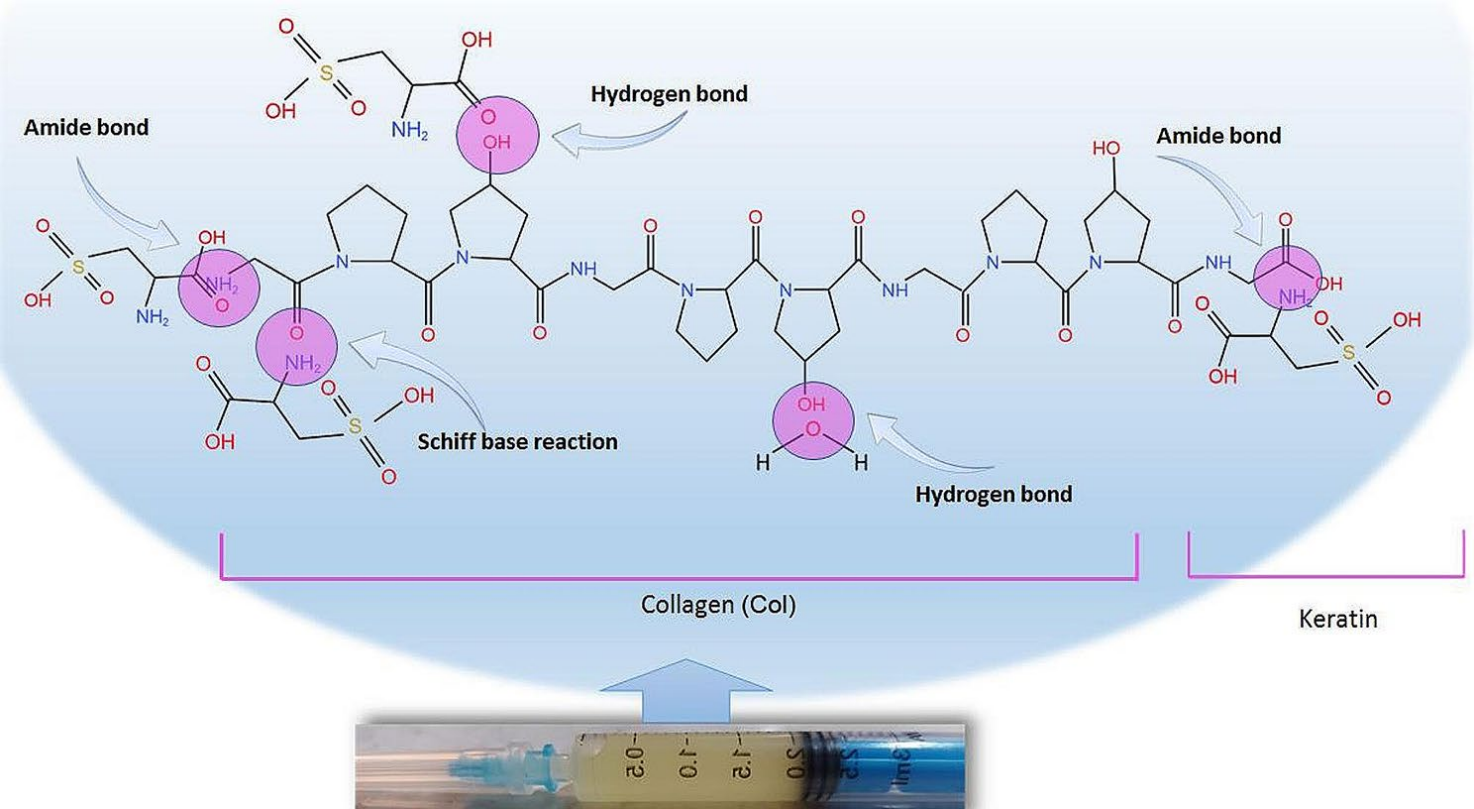
Interaction of Col with keratin after physical blending with possible mechanisms

In the current study, the myogenic properties of mouse C2C12 cell-laden Col/Keratin hydrogel were studied in a mouse model of biceps femoris injury. Elastic and rheological properties of the hydrogel are essential factors to be considered in injectable systems. These characteristics are strongly dependent on the chemical composition, cross-linking procedure, cross-linking agents, and their concentration [44]. In rheology investigations, the elastic and viscous behavior is evaluated by determining the frequency of shear force. Hydrogels with improved mechanical properties exhibit a higher frequency of swinging [45]. Type I Col fibers are well known for their stiffness (Young’s modulus of 1-800 MPa) and the ability to form viscoelastic networks (with elastic shear moduli of 1–200 Pa) [46]. Col can react with aldehydes and ketones with its several side chains and free carboxyl, amide, and hydroxyl groups, resulting in the formation of cross-links inside or outside of its molecular network. This cross-linking can lead to the production of a hybrid structure which improves the injectability and elastic properties of the Col networks (Fig. 6) [47]. In this study we incorporated human hair-derived keratin into the structure of type I Col. Molecular interactions and hydrogen bond between two polymers was proved in FTIR results according to the peak shifting in the amide I, amide II, and C-H area. In this study, Col/Keratin hydrogel exhibited viscous flow under shear stress (shear-thinning) which indicates ideal injectable properties for minimally invasive tissue engineering applications. The viscosity of both hydrogels at recommended temperatures was established to reduce with increasing the shear rate which revealed the shear thinning behavior of gels regardless of the temperature used. The high cysteine residue content of keratin leads to an increase in elasticity and a decrease in the viscous moduli of the polymer. On the other hand, according to its stable structure, keratin has been used in several tissue engineering applications such as volumetric muscle loss treatment [48]. As an instance, keratin hydrogels loaded with skeletal muscle progenitor cells/growth factors were implanted in the *latissimus dorsi* muscle of an established murine model with volumetric muscle loss injury. Results showed the overall effectiveness of keratin implants n muscle regeneration and a great recovery of contractile force was reported using keratin/growth factor hydrogels [49]. Also, due to its specific rheological properties, keratin has been considered a favorable lubricant agent for extrusion bio-inks in several 3D printing studies. Results confirmed the important role of keratin protein in increasing the extruding potential of the bio-ink without the need to use other rheological tuning agents [50]. Numerous studies have shown that keratin possesses appropriate biocompatibility and biodegradability which makes it a suitable natural substrate for tissue regeneration purposes [51–53]. Due to its excellent physical properties and resistance to enzymatic degradation, keratin-based hydrogels are suitable for long-term regeneration periods [54]. Besides, it has been shown that keratin has anti-inflammatory capacity and low-rate immunogenicity [55]. These features indicate that keratin-based substrates are suitable options for both research and therapeutic objectives [56, 57]. Strain stiffening properties in hydrogel systems is an interesting issue which is one inherent trademark of keratins that can considerably stiffen following increasing strain and in that way shelter cells and tissue from high deformation stress [58]. The strain stiffening of keratin arises from its hierarchical structure and the great extensibility of keratin strands allows the limited unfolding of subunits without strand rupture [59]. Our results discovered that the mixture of Col/Keratin hydrogel lost its strain stiffening capability but the decreasing rate of modulus was not intense during the initial stage of the test (near-linear) and G´, G´´ reached 33100,5270 Pa under 3.75% strain. However, it should be noted that, in this study, decreased stiffening of the hydrogel is favorable for extrudability of the hydrogel. This properties can be related to the fact that disulfide bonds of keratin was broken following oxidative extraction of keratin and its stiffness is dependent on the side chain chemical groups. It was previously revealed that, for keratin, the strands joining between thick bundles is fragile [60], this weak coupling results more strand slipping under forces and thus does not follow strain stiffening.

Data showed the maximum myogenic properties of Col/Keratin hydrogel when co-administrated with mouse myoblasts into the injured muscle tissue. We noted that the number of newly generated myofibers was increased in Col/Keratin + Cells groups compared to Col, Col/Keratin, and Col + Cell groups. Histological analysis indicated numerous immune cells with degrading hydrogel in the Col hydrogel alone group. Col has been extremely used in the fabrication of biomimetic materials in the context of tissue engineering [61]. Unfortunately, the *solo* application of Col is limited for the fabrication of hydrogels because of the high biodegradation rate and weak mechanical properties [62]. Here, the physical blending of Col with human hair keratin resulted in a slower degradation rate. One reason would be that keratin can provide thiol groups due to the existence of cysteine residues in its structure, leading to the formation of disulfide crosslinks with Col [63]. Besides, the existence of additional hydrogen bonds between the Col and keratin can in part, but not completely, increase hydrogel resistance to changes in environmental factors [64]. The formation of newly generated myofibers in groups that received Col/Keratin hydrogel alone or in combination with mouse myoblasts would be related to the development of more aligned Col fibers in the blend structure [65]. It is postulated that keratin is an intermediate filament-forming protein released by epithelial cells and triggers the formation of fibers in vivo [66]. These features can help the endogenous resident cells and exogenous transplanted cells to easily attach to fibers and acquire suitable morphologies in response to the signaling factors [67]. In support of this notion, we found more aligned newly generated myofibers in mice that received cell-free Col/Keratin hydrogel or the combination of Col/Keratin hydrogel and C2C12 myoblasts. In the Col group, newly generated cells are juxtaposed to each other with irregular patterns while this connection is regular and tight in Col/Keratin hydrogel groups. The existence of glutamic acid/aspartic acid/serine and leucine/aspartic acid motifs can induce cell attachment and proliferation in multiprotein hydrogels composed of Col and keratin with a ratio of 1:1 [68]. We indicated that co-transplantation of Col/Keratin and myoblasts promoted protein levels of MyoD, a factor associated with myogenic differentiation, at the site of injury. These data showed that Col/Keratin hydrogel alone or in combination with myoblasts can promote muscular tissue regeneration via the acceleration of muscle progenitor cell differentiation. Passipieri and colleagues indicated that the injection of factor-enriched or factor-free keratin hydrogels can promote neo-myogenesis in rats with volumetric muscle loss after 12 weeks [69]. They also indicated that keratin-based hydrogels increase muscle tissue regeneration via the stimulation of heavy chain myosin [69]. Despite the changes in the protein levels of MyoD, we did not find significant differences in heavy chain myosin contents between the experimental groups. One reason would be the current study was performed in over a short period 15 days whereas Passipieri and co-workers monitored heavy chain myosin contents after 12 weeks. In our study, we noted prominent immune cell recruitment to the site of injury after injection of Col hydrogel with a large number of CD31 endothelial cells, indicating angiogenic potential. It seems that the increased angiogenesis is due to active pro-inflammatory response and recruitment of immune cells to degrade Col fibers.

## Conclusion

In recent years, muscle tissue engineering has witnessed eminent technological advances in developing natural/synthetic substrates for the restoration of injured myocyte function in circumstances associated with several muscular degenerating diseases or volumetric loss. Here, it is suggested that the encapsulation and transplantation of myoblasts with Col/Keratin hydrogel promoted appropriate angiomyogenesis in a mouse model of biceps femoris injury without excessive immune system reactivity. It is thought that future studies can sophistically modify Col/Keratin hydrogel using different technologies for muscular tissue regeneration. The load of distinct cell types, growth factors, and other therapeutics can increase the regenerative outcomes.

### Electronic supplementary material

Below is the link to the electronic supplementary material.

Supplementary Material 1

## Data Availability

Data are available from the authors upon reasonable request from the corresponding author Dr. Reza Rahbarghazi.

## Abbreviations

Col: Collagen
DMEM: Dulbecco’s Modified Eagle Medium
ECM: Extracellular matrix
IHC: Immunohistochemistry
MyoD: Myoblast determination protein 1
PBST: Phosphate-buffered saline with Tween 20
PBS: Phosphate-buffered saline
PVDF: Polyvinylidene fluoride
KBr: Potassium bromide
SDS-PAGE: Sodium dodecyl-sulfate polyacrylamide gel electrophoresis
