# Supplementary material for: Multiprotein collagen/keratin hydrogel promoted myogenesis and angiogenesis of injured skeletal muscles in a mouse model
Source: BMC Biotechnol. 2024 Apr 26;24:23. doi: 10.1186/s12896-024-00847-4 (PMC11055224; doi:10.1186/s12896-024-00847-4)
Supplement: Supplementary file 1 — Supplementary Material 1 [file 12896_2024_847_MOESM1_ESM.pdf]

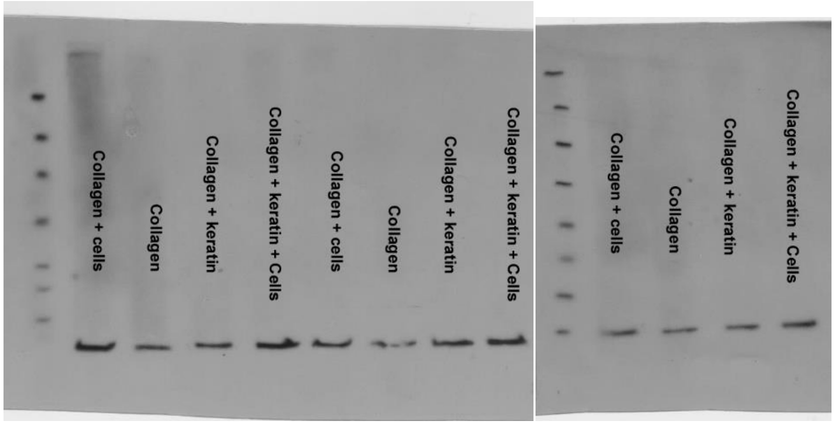

Myosin protein levels in different groups (n=3)

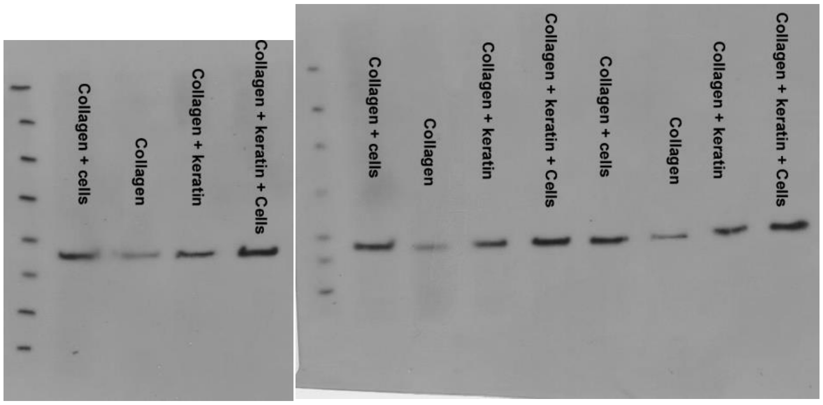

MyoD protein levels in different groups (n=3)

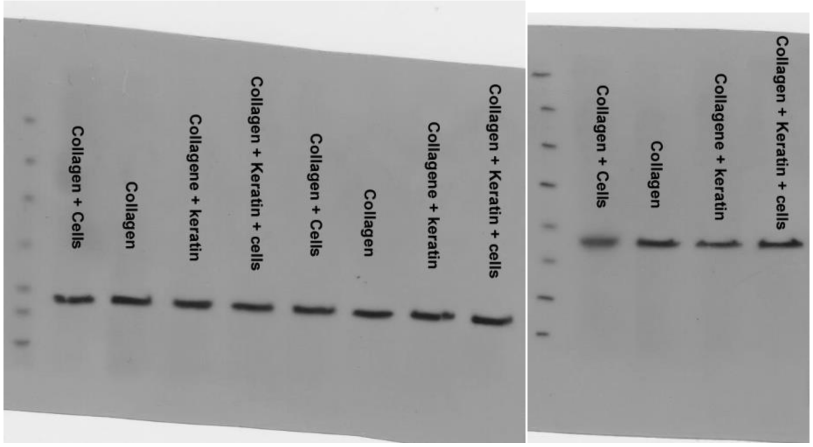

Beta-actin protein levels in different groups (n=3)

Figure legend. Westernblotting analysis of MyoD and myosin (*n*=3). Data revealed the induction of MyoD in Col + Cells and Col/kertain + Cells groups, indicating the maturation of muscle progenitor cells. Despite the increase of myosin in the Col/Kertain

+ Cells group, the differences were statistically non-significant. One-way ANOVA analysis with Tukey test. *$p<0.05$; ***$p<0.001$; and ****$p<0.0001$.
